# Organ donation in Brazil: individual autonomy versus family consent

**DOI:** 10.1590/1516-3180.2025.1441.17102025

**Published:** 2025-12-15

**Authors:** Gabriela Favaro Faria, Marcelo de Azevedo Granato, Paulo Manuel Pêgo-Fernandes

**Affiliations:** I Health Sciences, Escola de Enfermagem, Universidade de São Paulo (USP). Assistant Director, Discipline of Thoracic Surgery, Instituto do Coração (InCor), Hospital das Clínicas, Faculdade de Medicina, Universidade de São Paulo (HCFMUSP), São Paulo (SP), Brazil.; II Law, Universidade de São Paulo (USP), São Paulo (SP), Brazil; Università degli Studi di Torino, Turin, Italy. Professor, Centro Universitário FACAMP – Faculdades de Campinas, Campinas (SP), Brazil; Faculdade de Direito de Sorocaba (FADI, Sorocaba), Sorocaba (SP), Brazil.; III Vice-Director, Faculdade de Medicina, Universidade de São Paulo (FMUSP), São Paulo (SP), Brazil; Professor, Departamento de Cardiopneumologia, Faculdade de Medicina, Universidade de São Paulo (FMUSP), São Paulo (SP), Brazil; Director, Departamento Científico, Associação Paulista de Medicina (APM), São Paulo (SP), Brazil.

 Brazil has the largest public transplantation system worldwide. Nevertheless, approximately 3,000 people die annually while on the organ waiting list, highlighting the difficulty of balancing the growing demand with the limited donation rate.^1^

 The Brazilian Registry of Transplants (Registro Brasileiro de Transplantes – RBT), maintained by the Brazilian Association of Organ Transplantation (Associação Brasileira de Transplante de Órgãos – ABTO), is the primary tool for monitoring donation and transplant activities. In its 2024 report, the RBT found that the potential donor notification rate had reached 71 per million population (pmp), meeting the stipulated target (70 pmp). However, the effective donation rate remained below expectations (27% versus the target of 30%), primarily because of high family refusal (46%). Therefore, the rate of effective donors was 19.2 pmp, a rate lower than that recorded in 2023 (19.9 pmp) and below the projected target for 2024 (21 pmp).^1^

 Authorization for organ removal intended for transplantation has consistently provoked intense debate, as it concerns sensitive issues rooted in society’s core values and fundamental legal principles, such as human dignity, personality rights, and individual autonomy.^2^

 Brazil’s legislative evolution illustrates the difficulties in finding an adequate consent model. The first three laws regulating transplantation in Brazil, enacted in 1963, 1968, and 1992, adopted the informed consent model, in which organ removal depended on the explicit consent of the individual during their lifetime or, in its absence, on the authorization of their family members after death.^3-6^

 To increase the number of donations and transplantations, Law No. 9,434/1997 was enacted, establishing the presumed consent model, according to which all citizens would be considered donors unless a contrary declaration was registered in official documents.^7,8^

 However, the practical application of this measure proved counterproductive, as, instead of increasing the number of donations, it significantly raised refusal rates.^9^ Given this scenario, the mandatory aspect was revoked in October 2000.^10^ Subsequently, Law No. 10,211/2001 reinstated informed consent, attributing the final decision regarding donation exclusively to the family, even if the donor had expressed their decision during their lifetime.^11^

 Mechanisms to reinforce individual autonomy in organ donation have been proposed. One example is the Electronic Authorization for Organ, Tissue, and Part of the Human Body Donation (Autorização Eletrônica de Doação de Órgãos, Tecidos e Partes do Corpo Humano – AEDO), regulated by Provision No. 149/2023 of the National Council of Justice (Conselho Nacional de Justiça – CNJ).^12^ This instrument enables any citizen to register, during their lifetime, electronically and free of charge, the intention to donate organs, tissues, and body parts after death. The document is issued free of charge at notary offices, made accessible to authorized physicians, and may be revoked at any time, representing a symbolic and practical advance in registering an individual’s will. 

 This scenario reinforces the importance of discussing new consent models. The Federal Constitution, in Article 199, states that "the law shall provide for the conditions and requirements that facilitate the removal of organs, tissues, and human substances for transplantation purposes."^2^ Thus, the legislation regulating the topic has the duty to facilitate the donation process, which Law No. 9,434/1997, in its current form, does not do.^7^

 The draft reform of the Civil Code proposed relevant changes in the direction of the constitutional mandate. The proposal amends Article 4 of Law No. 9,434/1997, establishing that "the removal of tissues, organs, and body parts from deceased persons, for transplantations or other therapeutic purposes, shall not require authorization from any family members when the deceased has determined in writing, or has recorded in any of their personal documents, explicit authorization for the donation."^13^

 Furthermore, the draft provides for the amendment of Article 14 of the Civil Code, which states that "the gratuitous disposition of one’s own body, in whole or in part, after death, is valid for scientific or altruistic purposes. Sole Paragraph. The act of disposition may be freely revoked at any time." In the draft, this article gains an additional paragraph: "§1^º^ – If there is a written disposition by the individual themselves, family authorization is not required, and in its absence, it shall be given according to the order of legitimate succession."^14^

 International experiences reinforce the fact that no single consent model can guarantee success. In Spain, where presumed consent has been in force since 1979, a significant increase in postmortem donations occurred only after the creation of the national network of hospital transplant coordinators in 1989.^15^

 In various Latin American countries that have adopted presumed consent, there has been no significant effect on donation rates. In the United States, informed consent is associated with high donation rates owing to the strengthening of highly professionalized organ procurement organizations with clear goals.^16^

 There are also alternative models that preserve the family’s decision but offer additional incentives. One example is the prioritization, on waiting lists, of patients with first-degree relatives who donated organs after death. Thus, family members who must decide on the donation of their deceased relatives’ organs would have an additional incentive to approve the donation. This measure, adopted in Israel, resulted in a significant increase in family authorizations.^17^

 This debate reinforces the idea that organ donation legislation must align with societal values and effective public policies. Law No. 14,722/2023, which established the National Policy for Awareness and Incentive for Organ and Tissue Donation and Transplantation, emphasizes the importance of public discussion, education, and demystification of the topic.^18^ Many family refusals stem from misconceptions, negative experiences in hospital care, or delays in organ procurement. 

 Therefore, the future of organ donation in Brazil requires not only legislative revisions but also strengthening of the National Transplant System, quality information, and societal engagement. The AEDO and reform of the Civil Code represent relevant advances, but only a balanced combination of respect for individual autonomy, care for families, and social awareness will consolidate the true culture of donation in the country. 
